# GATA2 Related Conditions and Predisposition to Pediatric Myelodysplastic Syndromes

**DOI:** 10.3390/cancers12102962

**Published:** 2020-10-13

**Authors:** Antonella Bruzzese, Davide Leardini, Riccardo Masetti, Luisa Strocchio, Katia Girardi, Mattia Algeri, Giada Del Baldo, Franco Locatelli, Angela Mastronuzzi

**Affiliations:** 1 Department of Hematology/Oncology, Cell and Gene Therapy, IRCCS Bambino Gesù Children’s Hospital, 00165 Rome, Italy; luisa.strocchio@opbg.net (L.S.); katia.girardi@opbg.net (K.G.); mattia.algeri@opbg.net (M.A.); giada.delbaldo@opbg.net (G.D.B.); franco.locatelli@opbg.net (F.L.); angela.mastronuzzi@opbg.net (A.M.); 2 Pediatric Hematology/Oncology, Sant’Orsola Malpighi Hospital, University of Bologna, 40138 Bologna, Italy; davide.leardini3@studio.unibo.it (D.L.); riccardo.masetti5@unibo.it (R.M.); 3 Department of Maternal Infantile and Urological Sciences, Sapienza University of Rome, 00161 Rome, Italy

**Keywords:** myelodysplastic syndromes, cancer predisposition, *GATA2* deficiency, childhood MDS, pediatric cancer

## Abstract

**Simple Summary:**

*GATA2* deficiency is considered one of the most common cancer predisposition syndromes determining myelodysplastic syndrome in children. Little is known of this recently described syndrome, often resulting in a misdiagnosis and unclear management. In this review, we describe *GATA2* deficiency clinical presentation in order to focus on phenotypes that, in patients with myelodysplastic syndrome, may be suggestive of *GATA2* deficiency. Moreover, due to the lack of clear guidelines, we performed an overview on literature data regarding management of *GATA2*-related myelodysplastic syndrome, in order to understand the best choice of treatment for these patients.

**Abstract:**

Myelodysplastic syndromes (MDS) are hematopoietic disorders rare in childhood, often occurring in patients with inherited bone marrow failure syndromes or germinal predisposition syndromes. Among the latter, one of the most frequent involves the gene GATA binding protein 2 (*GATA2*), coding for a transcriptional regulator of hematopoiesis. The genetic lesion as well as the clinical phenotype are extremely variable; many patients present hematological malignancies, especially MDS with the possibility to evolve into acute myeloid leukemia. Variable immune dysfunction, especially resulting in B- and NK-cell lymphopenia, lead to severe infections, including generalized warts and mycobacterial infection. Defects of alveolar macrophages lead to pulmonary alveolar proteinosis through inadequate clearance of surfactant proteins. Currently, there are no clear guidelines for the monitoring and treatment of patients with *GATA2* mutations. In patients with MDS, the only curative treatment is allogeneic hematopoietic stem cell transplantation (HSCT) that restores normal hematopoiesis preventing the progression to acute myeloid leukemia and clears long-standing infections. However, to date, the donor type, conditioning regimen, and the optimal time to proceed to HSCT, as well as the level of chimerism needed to reverse the phenotype, remain unclear highlighting the need for consensus guidelines.

## 1. Introduction

Myelodysplastic syndromes (MDS) are clonal hematopoietic disorders, characterized by cytopenia of one or more hematopoietic lines, ineffective hematopoiesis, and a possible evolution to acute myeloid leukemia (AML). Sporadic MDS is primarily an elderly disease with an incidence of 75 per 100,000 in patients older than 65 years [1], while, during childhood, MDS are rare with an annual incidence of 1–4 cases per million. MDS account for less than 5% of all childhood hematologic malignancies, and many childhood MDS occur in the contest of germinal predisposition syndromes or Inherited Bone Marrow Failure Syndromes (IBMFS) [2].

Among germline predispositions, one of the most frequent involves the gene GATA binding protein 2 (*GATA2*). The first description of *GATA2* mutation dates back to 2011, when four independent groups described four clinical phenotypes: Emberger syndrome (lymphedema and monosomy 7), MonoMAC syndrome (monocytopenia and Mycobacterium avium complex infection), dendritic cell, monocyte, B and natural killer (NK) lymphoid deficiency (DCML), and familial MDS/AML, all associated with a *GATA2* deficiency [3,4,5]. The first description of *GATA2* related MDS is due to Scott and colleagues, reporting the first description of *GATA2* germline mutation in four MDS/AML families. They observed in these families the missense mutations T354M, and the 355delT mutation, absent in 659 healthy controls, such as any other variants of the *GATA2* coding sequence. These mutations involve highly conserved, five consecutive threonine residues in the zinc finger 2 domain (ZF2) of the GATA2 protein, responsible for DNA-binding and homodimerization [6]. In 2016, the cooperative European Working Group on Childhood MDS (EWOG-MDS) consortium conducted a large study analyzing 508 young patients (children and adolescents), 426 with primary MDS, and 82 with secondary MDS enrolled in the prospective studies EWOG-MDS 98 and EWOG-MDS 2006 over a 15-year period. Germline *GATA2* mutations were found in 7% of primary MDS cases, 15% of advanced MDS, and were never found in children with MDS secondary to aplastic anemia or previous cancer therapy. *GATA2* mutations were more prevalent in advanced MDS rather than refractory childhood cytopenia; moreover, *GATA2* mutated patients were older at diagnosis and presented more often with advanced MDS and monosomy 7 compared to *GATA2* wild type patients [7]. These data have pointed the attention to this unique *GATA2* related-MDS, but, even if the comprehension of the disease has progressed, only a few recommendations are available about the best clinical management.

## 2. Biological Features

*GATA2* is a key transcriptional regulator of hematopoiesis involved in the development and maintenance of the stem cell pool through hematopoietic stem cell (HSC) survival and self-renewal. It is also involved in the development of monocytes, mast cells, NK cells, and megakaryocytes [8]. Even if the human syndromes related to *GATA2* deficiency have been described only recently, the role of *GATA2* in leukemogenesis has long been studied. This protein contains two ZF domains and a nuclear localization signal. *GATA2* mutations involving the two ZF domains can be distinguished by phenotypic correlations and mutational clustering suggesting different leukemogenic mechanisms [9]. *GATA2* binds to the consensus sequence *W/GATA/R (W 5 A or T and R 5 A or G)* in promoter/enhancer regions of target genes such as *RUNX*, *TAL1*, *FLI1*, *SPI1 (PU.1)*, *LMO2*, regulating during embryogenesis the transformation of endothelial cells into hematopoietic cells, the formation of HSCs, and the definitive hematopoiesis [10].

Today, nearly 150 *GATA2* mutations have been described, as either germline or somatic mutations. Roughly one-third of *GATA2* germline mutations are inherited, while two thirds occur de novo. A wide range of mutations were described: in-frame insertions or deletions; single nucleotide variants arising amino-acid substitution in the exons 3, 4 and 5, encoding the two ZF domains; a small number of non-sense or frame-shift mutations and whole gene deletions (distributed across the ZF2 domain); splice site mutations were also described between exons 3 and 4; discrete mutations of the intron 5 enhancer have also been reported. Overall, in about two-thirds of cases reported, mutation involve the ZF domains. No mutations have been reported in the 5′ or 3′ untranslated regions, nor in the distal section of the last exon, beyond the region encoding the ZF2 domain.

**Figure 1:**
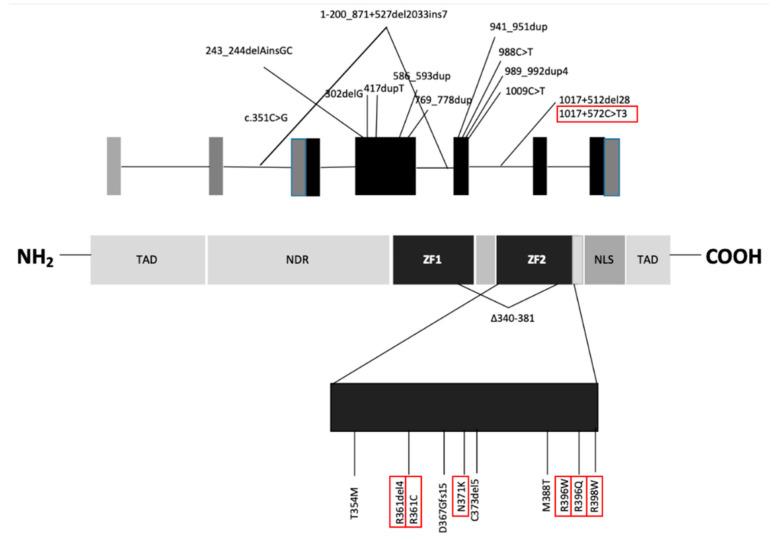
*GATA2* gene and protein structure. *GATA2* gene and protein structure. Mutations most frequently associated with MDS are circled [7]. TAD: transactivation domain, NRD: negative regulatory domain, ZF: zinc-finger domains, NLS: nuclear localization signal.

More than half of the variants described are single amino-acid substitutions that may result in the translation of mutated protein with an altered function. Gene deletions and frame-shift mutations are null alleles that may lead to the same phenotypes. Many single amino-acid substitutions impair the DNA-binding function of the ZF domains, making the protein functionally inactive [11]. However, it is also possible that the mutated protein maintains a residual function or can even act in a dominant negative fashion, as reported for T354M, or can result in a gain of function, as reported for the L359V variant, shown in two cases of blast transformation of chronic myeloid leukemia (CML) [4,12]. There are no known germ-line mutations affecting ZF1, suggesting that these kinds of mutations may be embryo-lethal [9]. Of all the known regulatory regions of *GATA2*, germline mutations have been reported only in the intron 5 enhancer [13]; recently, a new mutation (*c.351C>G*) was described in the intron 3 leading to an aberrantly spliced *GATA2* transcript with a 136-bp internal deletion [14]. The most common *GATA2* mutations are shown in Figure 1. Wlodarski et al. have found that *GATA2* mutations can also be associated with cytogenetic abnormalities such as monosomy 7 and trisomy 8. Overall, the prevalence of *GATA2* mutations reach 72% in adolescent (age 12–19) with MDS associated with monosomy 7, while its prevalence is 16% in patients with MDS and trisomy 8. Also, *der (1;7)* has been described in patients with MDS carrying *GATA2* mutation [7].

## 3. Clinical Features

*GATA2* germline mutations are associated with a wide range of signs and symptoms. The proportion of asymptomatic patients carrying *GATA2* germline mutations has been estimated to be 38% by age 20 and 8% by age 40. The median age at onset of the first symptoms is 18 years [15]. Among symptomatic patients, the symptoms can be extremely variable [5,8]. The most frequent clinical phenotypes are summarized below and in Table 1.

### 3.1. Oncological and Non-Oncological Hematological Abnormalities

In patients carrying *GATA2* germline mutations, the risk of developing MDS/AL is 6% at the age of 10 years, 39% at the age of 20 years, and 81% at the age of 40 years. MDS are the most common hematological malignancies and can evolve into AML. Cases of T-cell acute lymphoblastic leukemia (in addition to monosomy 7) and juvenile myelomonocytic leukemia were also described [7,15].

Patients without hematological malignancies often show abnormal blood count with neutropenia, thrombocytopenia, and macrocytosis [15]. The mononuclear cells profiling reveals monocytes, dendritic cell, B cells, and NK deficiency [17], even though Wlodarski and colleagues described more cases of monocytosis than monocytopenia [7]. Analyzing bone marrow and peripheral blood in flow cytometry, the most distinctive feature of *GATA2*-related MDS seems to be the reduction of B cells, compared to healthy controls, aplastic anemia, and MDS patients without *GATA2* deficiency. *GATA2*-deficient patients could also present a relative increase in marrow T cells with an inverted CD4:CD8 ratio and a higher proportion of plasma cells, sometimes with an atypical phenotype [18,19]. Bone marrow biopsy performed in patients without hematological malignancies often show atypical megakaryocytes that can be larger and abnormal with separated nuclear lobes, and smaller with separated nuclear lobes or micromegakaryocytes, even in the absence of overt MDS. Additionally, patients with non-tuberculous mycobacterial infections (NTM), granulomata, and mycobacteria could be found in the marrow [20]. In pediatric patients with MDS, a lack of B cells and B-cell progenitors is suggestive for GATA2 deficiency [18,19].

### 3.2. Infections

Patients with germline *GATA2* mutation present a wide range of immune dysfunctions, leading to severe infections, primarily generalized warts and mycobacterial infection. At the age of 20, the cumulative rate of bacterial infection is 33% and at the age of 40 years is 64% [15].

This high rate of infections is related to immune defects of cell mediated and humoral immunity. The impaired T-cell mediated immunity leads to recurrent respiratory tract infection; the impaired viral clearance and the defective immunosurveillance are responsible for the higher rate of malignant transformation of HPV neoplasm. Related HPV is the most common viral infection, occurring in about two-thirds of *GATA2* germline mutation carriers, presenting with warts, condyloma, and/or dysplasia. When warts are associated with monocytopenia, a *GATA2* deficiency must be suspected. The lack of dendritic cells impairs recognition of viruses and intracellular pathogens contributing to disseminated herpes virus infections and mycobacterial susceptibility. Furthermore, it has been described that in the site of infection, tissue macrophages are present but organized granulomatous inflammation is defective [8,15,16].

### 3.3. Pulmonary Alveolar Proteinosis (PAP)

Defects of alveolar macrophages led to PAP due to inadequate clearance of surfactant proteins. Patients with *GATA2* mutation who develop a PAP do not have antibodies anti-GM-CSF and respond poorly to lavage and GM-CSF therapy.

Patients without PAP can present abnormal pulmonary function such as diffusion or ventilatory defects (obstruction, restriction, or a mixed pattern) and/or structural abnormalities identified on chest CT including reticular opacities, nodules, ground glass opacities, crazy paving para-septal emphysema, and subpleural blebbing. Alveolar macrophages are abundant in bronchoalveolar lavage fluids [16].

### 3.4. Cardiovascular and Lymphatic

Chronic lymphedema is a common feature that could be either unilateral or bilateral, involving lower extremities and genitalia. Mouse models suggest that an intact *GATA2* function is required for proper lymphatic vascular development and lymphatic valve morphogenesis [21]. Furthermore, *GATA2* is involved in vascular integrity and cell adhesion, as suggested by its high expression in endothelial cells [22]. Lymphedema, particularly in an adolescent or young adult with cytopenia, is highly suggestive of *GATA2* deficiency.

### 3.5. Other Oncological Malignancies

The majority of solid tumors are related to underlying viral infections, in particular HPV-related dysplasia and EBV-related tumors, such as leiomyosarcoma. Other cancers reported are renal cell carcinoma, pancreas adenocarcinoma, breast cancer, locally invasive desmoid tumor of the chest wall and Merkel cell carcinoma in a contest neurofibromatosis type 1 [16,23].

### 3.6. Deafness

Sensorineural hearing loss often occurs in patients with whole gene deletions of *GATA2*. Haugas and colleagues showed that, in mouse models, *GATA2* is involved in ear morphogenesis. Without *GATA2*, the semicircular ducts fail to grow to their normal size, and the surrounding mesenchymal cells are not removed properly to generate perilymphatic space [24].

## 4. Phenotype-Genotype Clustering

As previously mentioned, there are more than 150 known mutations in *GATA2*-related disorders, but there is no evidence of a clear genotype–phenotype correlation. However, it has been noted that certain phenotypes tended to cluster within families. It has been observed from analyzing the association between phenotype and the type of *GATA2* mutation that severe infections are more frequent and have an earlier onset in patients with *null* mutations. Lymphedema has only been observed in patients with *null* mutation or regulatory mutations [16]. In a survey performed among the French and Belgian patients with *GATA2* mutation, the investigators found a significantly higher risk of developing leukemia in the group with missense mutations compared to the group with a non-sense or frameshift mutations [15].

In 2015, Mir and colleagues reported an association between missense mutation with a high risk of developing MDS/AML, while deletion was associated with dysmorphic features, monocytopenia, and high risk of infections [25].

The relation between genetic lesion and different clinical presentation could be explicated by the different *GATA2* regulation in the biological systems; it has been noted that genetic lesions affecting certain regions of *GATA2* are related to defects of certain organs and systems rather than others. Moreover, *GATA2* instigated genetic networks that vary in different cellular contexts and in a stress state rather than a steady state [26]. The mechanisms sustaining the development of MDS in *GATA2* deficiency are not completely understood. Clonal evolution could be associated with the development of cytogenetic clones and progression to MDS/AML. The most common somatic mutations in hematopoietic malignant clones involve the gene *ASXL1*, reported in 29% of case series [27]. Other somatic mutations have also been reported, including *SEPTBP1*, *STAG2*, *RUNX1*, *CBL*, *EZH2*, *NRAS*, *JAK3*, and *PTPN11* [28]. It has been noted that *GATA2* mutation is associated with a high elevation of FLT3 ligand that progressively increase with the evolution of cytopenia and clinical complications, suggesting a possible usage in clinical monitoring [17]. In vitro studies showed that *GATA2* promotes the transcription and expression of IL1b and CXCL2 with positive feedback on an RAS/MAPK-GATA2-IL1b/CXCL2 axis. High expression of CXCL2 promotes leukemic cell proliferation and correlates with a poor prognosis of patients with AML [29].

**Figure 2:**
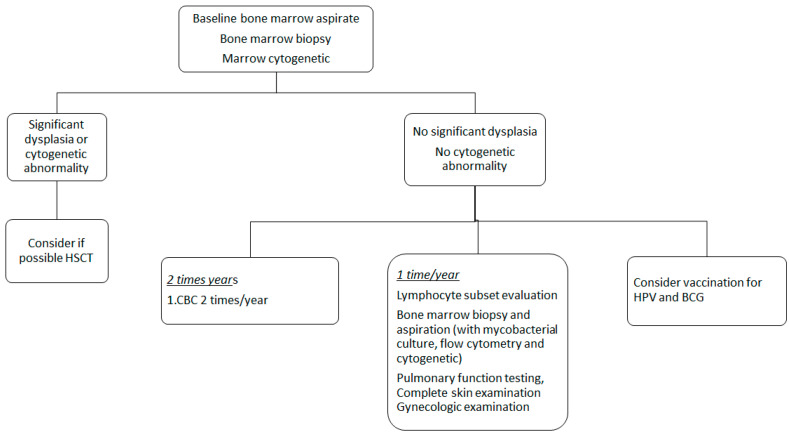
Possible algorithm for management of patients with *GATA2* deficiency.

In a recent report published in 2018 by McReynolds, the authors selected 25 subjects (probands or parents) with a known *GATA2* deficiency and performed a panel sequence of 49 genes commonly mutated in AML or MDS. Variants were found in 73% (8/11) of patients with MDS, 71% (5/7) of patients with *GATA2* deficiency related bone marrow and immunodeficiency disorder (G2BMID), and only in one subject with normal blood cells count and bone marrow histology. These additional somatic mutations affected genes involved in trascriptional regulation, DNA modification, chromatin regulation, cell–cell or cell-matrix cohesion, and cell signaling. The most frequent somatic mutations involve *ASXL1*, that were seen in 6 of 18 patients with MDS or G2BMID (33%). In the MDS group, another frequent mutation involved the gene *STAG2*, seen in 3 of 11 MDS patients. Most patients had normal bone marrow cytogenetics (68%, 17/25) (Figure 2). The most common abnormality in this cohort was isolated trisomy 8 (5/8), in contrast to another report that describes an association with monosomy 7 [7,30].

Concerning the differences among races, it should be noted that most of literature reports concerned the Caucasian race. Few pieces of data are available about *GATA2* germline mutation in Asiatic patients; in particular, one case report described a Japanese patient with a *GATA2* mutation c.988C>T affected by immunodeficiency, who developed MDS [31]; recently, a case series of pediatric patients with hematological malignancies with monosomy 7 and germline *GATA2* mutation have been described [32]. In a study performed in Taiwan on de novo AML, the authors found a *GATA2* mutation in 43 patients; most were missense mutations, as reported in the most study on Caucasians [33]. These data were confirmed in the cohort study reported by Spinner and colleagues, in which 54% of patients were Caucasian, with no differences in the type of genetic abnormalities among Caucasians, Hispanics, African Americans, and Asians [16].

## 5. Management

Currently, there are no clear guidelines for the monitoring and treatment of patients with *GATA2* mutations. The first critical issue is the diagnosis of the *GATA2* deficiency. Although an exceedingly high number of mutations in the *GATA2* locus have been described, the complete coverage of the *GATA2* gene is technically cumbersome. Some authors suggest that, when disease is highly suspected and the sequencing of exons and intron 5 is negative, a copy number variation analysis and mRNA sequencing should be performed [28].

In patients with MDS, a *GATA2* deficiency should be suspected in case of suggestive clinical features, such as monocytopenia, NTM infections, recurrent HPV infections, and lymphedema; the report of the European Working Group of MDS in children and adolescent patients with a high-risk MDS (often presenting other cytogenetic abnormalities) should be considered for *GATA2* deficiency [7]. Screening tests searching for healthy carriers should be performed in families with almost one case of *GATA2* deficiency; however, the best management for healthy carriers is not well established.

Once identified as a healthy carrier, experts suggest performing a baseline bone marrow aspirate and cytogenetic to identify *GATA2* related alterations. There is no complete agreement in the indications for bone marrow biopsy; however, most international societies including EWOG-MDS recommend this evaluation at diagnosis. Complete blood count with differential should be evaluated at least twice yearly, while lymphocyte subset assessment, bone marrow aspiration (with flow cytometry, cytogenetic and mycobacterial culture), pulmonary function testing, complete skin examination, and gynecologic examination should be performed once yearly [20].

Some authors suggest daily azithromycin prophylaxis for NTM, but clear guidelines for antibiotic prophylaxis are missing. Routine childhood vaccinations should be performed, including an HPV vaccination such as BCG vaccination. Patients with normal blood counts seem to tolerate these vaccinations well, and no data are available about tolerance of live virus vaccinations in patients with cytopenia [20]. BCG is generally well tolerated, but data available in literature are very limited and thus there is no agreement in recommending it [34].

Good oral hygiene and regular dental visits are recommended, especially for HPV-related oral disease surveillance, as well as periodic gynecological visits for women with *GATA2* deficiency [20].

Recommendations are shown in Figure 2.

In *GATA2* mutation, carriers who develop MDS, allogeneic hematopoietic stem cell transplantation (HSCT) represent the only curative option. HSCT restores normal hematopoiesis, resolves MDS (or AML), and clears long-standing infections. Moreover, despite the limitation of data, HSCT has been described in clinical cases associated with a regression of PAP and pulmonary hypertension [35] and the resolution of condylomas and cervical cancer in situ [36,37].

Currently, the best choice of conditioning regimen, donor source, and graft-versus-host disease (GVHD) prophylaxis remains unclear. Regarding the best time to proceed to HSCT, both myeloablative and reduced intensity conditioning regimens have been successfully used as well as different donor types: peripheral blood stem cells from matched related or unrelated donors, bone marrow stem cells from matched or haploidentical donor, and umbilical cord blood [20,37]. However, HSCT remains challenging because of many comorbidities presented in the *GATA2* related syndromes, such as the previously mentioned infections from *Mycobacterium avium* complex and PAP.

It is important to note that a myeloablative conditioning regimen followed by HSCT is the standard approach in pediatric patients with myelodysplasia, particularly in patients with high-risk features such as monosomy 7 (often found in patients with *GATA2* related MDS). Otherwise, patients with *GATA2* deficiency present variable grade of immunodeficiency and other comorbidity that could threaten the outcome of HSCT with a high rate of transplant related toxicity. Interestingly, different case series reported thrombotic complications after HSCT in *GATA2* deficient patients, sometimes with a fatal outcome [36,38,39].

In the analysis of MDS in children by the European Working Group, 34 *GATA2* mutation carriers have been transplanted. In this group, the 5-year OS (66% vs. 69%) and EFS (60% for both) after HSCT were comparable to a *GATA2* wild type group. The relapse rate, TRM, and the rate of infectious complications did not significantly differ between both groups. Specifically, the rate of infectious complications (cytomegalovirus reactivation, cytomegalovirus disease, Epstein–Barr virus infections/post-transplant lymphoproliferative disorder, adenovirus infections, viral, bacterial, fungal, and parasitic infections) was 66% in the *GATA2* deficient patients and 61% in the control group [7].

Hofmann et al. analyzed a cohort of 15 pediatric patients with *GATA2*-related MDS undergoing HSCT after a myeloablative conditioning regimen. They compared this cohort of patients to patients with a BMF/MDS and patients with acute leukemia, and found a comparable rate of graft failure, graft versus host disease, and transplant related mortality. Interestingly, patients with *GATA2* deficiency showed a higher incidence of neurological complication and thrombotic events [39], questioning the safety of myeloablative condition regimen in *GATA2* deficient patients.

Considering the ideal time to proceed to HSCT, a better prognosis was seen if HSCT was performed in an early phase without evidence of disease progression, but the EWOG-MDS 2017 guidelines on MDS- refractory cytopenia of childhood (RCC) recommended watchful waiting in case of absence of high-risk genetic alterations and stable blood counts. Therefore, it is not possible to state if HSCT is recommended in patients with *GATA2* deficiency without MDS with high-risk features. In the analysis performed by EWOG-MDS working group, a stable disease was rarely reported in *GATA2*-related MDS. Among the 57 children with *GATA2*-related MDS, seven patients did not undergo allogenic stem cell transplantation, three of these patients with stable, non-transfusion dependent RCC remained alive and without severe infections 5 to 16 years from diagnosis, the other four patients died for disease progression or infection after AML-like chemotherapy [7]. These data support the idea that a watch and wait strategy could not be safe in patients with *GATA2* related MDS.

Different managements of treatment have been described in literature, considering the presence or not of additional cytogenetic or molecular abnormalities, hematological features, and other clinical presentation. A brief summary is reported in Table 2.

Evaluating risks and benefits of both strategies (to transplant or not to transplant) in patients with stable disease, it could be suitable to distinguish *GATA2* deficient patients who undergo HSCT for bone marrow failure or immunodeficiency from patients who undergo HSCT for high-risk MDS or acute leukemia.

Some authors suggested the use of a non-myeloablative regimen for patients transplanted earlier with a proliferative disadvantage of the bone marrow and the use of a higher dose regimen when either the development of clonal progression has occurred or the patient has a hypercellular marrow [40].

However, to date, the donor type, the conditioning regimen, and the optimal time to proceed to HSCT as well as the level of chimerism needed to reverse the phenotype remain unclear [41], underlining the need for consensus guidelines. It is important to note that potential family donors before stem cell donation should be tested for *GATA2* abnormalities. In the United States, there is an ongoing clinical trial of the National Institute of Health Clinical Center with the aim to determine whether an allogeneic HSCT approach results in engraftment and restores normal hematopoiesis by day + 100 in patients with *GATA2* mutations (ClinicalTrials.gov Identifier: NCT01861106).

## 6. Conclusions

In summary, the somatic mutation landscape significantly differs between children and adults with MDS. A peculiarity of childhood MDS is represented by the frequent association with genetic (i.e., germinal) predisposition or with IBMFS. Specifically, with the increasing use of molecular test in clinical practice, more children diagnosed with MDS are likely to be diagnosed as having a genetic predisposition syndrome. Since advanced MDS and monosomy 7 are highly overrepresented in *GATA2*-related MDS, we suggest that monosomy 7 could be used as a diagnostic indicator for *GATA2* deficiency in adolescents, given the high prevalence of mutations in this subgroup of age. In particular, *GATA2* analysis has to be included in the workup of all young adults and children above the age of four diagnosed with MDS associated with monosomy 7, trisomy 8, or der(1;7), regardless of the presence of a clinical phenotype suggestive to *GATA2*-deficency or the family history. For children with *GATA2* mutations and MDS, the ideal time for HSCT seems to be during the hypo cellular phase of the disease and before serious complications (i.e., invasive infections) or progression to advanced MDS occur.

## Figures and Tables

**Table 1:** Clinical features of *GATA2* deficiency.

Clinical Features	Frequency (%)
Hematological features	
MDS	70–84 [15,16]
AML	14–19 [15,16]
ALL	1.3 [15]
AA	2.5 [15]
JMML	1.3 [15]
Immunodeficit	
Monocytopenia	49–78 [15,16]
B lymphopenia	86–100 [15,16]
NK lymphopenia	7.8–82 [15,16]
Neutropenia	47 [16]
Infections	
Severe viral infections	70 [16]
Disseminated NTM infections	53 [16]
Severe bacterial infections	49–56 [15,16]
Severe fungal infections	16% [16]
Persistent EBV viremia	11% [16]
Warts	
HPV-related	35–40 [15,16]
Oncologic	3.8 [15]
Lymphedema	11–15 [15,16]
Pulmonary features	
PAPs	3.8–18 [15,16]
Recurrent bacterial infections	56 [15]
Pulmonary hypertension	<20% [16]
Thrombotic complications	9–25 [15,16]
Deafness	1.3 [15]
Autoimmune features	11 [15]
Urinary tract malformation	5 [15]
Obsetrian complications	6.3–33 [15,16]
Hypothyroidism	1.3–14 [15,16]

**Table 2:** Cases described of *GATA2* related MDS/AML. * patients with GATA2 synonymous mutations.

No. of Patients with GATA2 Related MDS/AML	MDS Type	Additional Cytogenetic Abnormality	Median Age at MDS Diagnosis (Range)	HSCT	Outcome	Reference
3	AML(33%) MDS (66%)	t(1;21) (33%)	27.7 (10–38)	3/3	Relapse (33%) Alive (66%)	Mir, 2015 [25]
28	MDS-RAEB-1 (7%) MDS-RAEB-2 (4%) MDS-RCMD (89%)	Monosomy 7 (14%) Trisomy 8 (25%) Der(1;7) (4%)	35.4 (12–73)	*n*.a.	*n*.a.	Ganapathi [19]
7	MDS-RCMD (29%) MDS-RAEB-2 (29%) AML (43%)	Trisomy 8 (14%)	16.8 (13–25)	1/7	Alive (57%) Dead (43%)	Churpek [42]
5	MDS *n*.s. (80%) MDS-RCMD (20%)	Trisomy 8 (60%) Monosomy 7 (40%) Der(1;7) (40%)	*n*.a.	4/5	Alive (60%) Dead (40%)	Wang [43]
5	Marrow failure (100%)	Trisomy 8 (40%)	16.0 (12–22)	1/5	*n*.a.	Zahng [44]
57	RCC (54%) RAEB (35%) RAEB-t (11%)	Monosomy 7 (68%) Trisomy 8 (9%) Der(1;7) (7%)	12.0 (3–19)	50/57	Died (28%) Relapse (5%) Alive (67%)	Wlodarski [7]
11	RCC (73%) RCMD (9%) RAEB (18%)	Monosomy 7 (73%) Trisomy 8 (18%)	14.7 (4–21)	9/11	Alive (73%) Dead (27%) Relapse (9%)	Novakova [18]
5	MDS *n*.s. (100%)	*n*.a.	26.0 (7–60)	*n*.a.	*n*.a.	Schlums [45]
5	RCC (100%)	Monosomy 7 (80%)	9.8 (5–15)	5/5	Alive (80%) Dead (20%)	Fisher [46]
11	MDS *n*.s. (100%)	Trisomy 8 (45%)	33.5 (23–53)	*n*.a.	*n*.a.	McReynolds [30]
8 *	RCC (75%) RAEB (13%) MDS-MLD (13%)	Monosomy 7 (50%)	11.6 (3–24)	6/8	Alive (88%) Dead (13%)	Kozyra [47]
3	AML (33%) MDS (66%)	*n*.a.	19.0 (13–27)	2/3	Alive (66%) Dead after HSCT (33%)	Bogaert [36]
1	MDS *n*.s. (100%)	Monosomy 7 (50%)	22 (19–25)	1/2	Alive (50%) Dead (50%)	Fox [48]
6	AML (50%) RAEB (17%) RCC (33%)	Monosomy 7 (100%) Trisomy 8 (17%)	10.5 (5–15)	6/6	Alive (83%) Dead (17%)	Yoshida [33]
